# Influence of the incorporation of titanium dioxide nanofibers net on bond strength and morphology of a total etching adhesive system 

**DOI:** 10.4317/jced.60729

**Published:** 2023-09-01

**Authors:** Celso-Afonso Klein-Junior, Roberto Zimmer, Fábio-Herrmann Coelho-de-Souza, Henrique Cantarelli, Maruí-Cezar-Ribeiro-da Rosa Júnior, Eduardo-Galia Reston

**Affiliations:** 1 Lutheran University of Brazil, Canoas, Brazil; 2 Feevale University, Novo Hamburgo, Brazil; 3 Federal University of Rio Grande do Sul, Porto Alegre, Brazil

## Abstract

**Background:**

The aim of this study was to evaluate the nanoleakage and microtensile bond strength (μTBS) of an ethanol based-adhesive containing Titanium dioxide (TiO2) nanofibers to dentin.

**Material and Methods:**

TiO2 nanofiber was produced by electrospinning and it was inserted in an ethanol-based adhesive in 0.5, 1.5 and 2.5% by weight. The original adhesive did not receive nanofiber. The middle dentin was exposed by diamond saw under water-cooling and dentin was polished with wet 600-grit SiC abrasive paper. Resin composite build-ups were applied incrementally to the dentin after adhesive application. After storage in distilled water (24 hours/37°C) the teeth were sectioned perpendicularly to the bonded interface and sticks were obtained. Twenty-five sticks per group were tested by μTBS with a crosshead speed of 0.5mm/minute. The average values (MPa) obtained in each substrate were subjected to one-way ANOVA (α=0,05) with the tooth being considered the experimental unit. The nanoleakage pattern was observed in ten sticks per group and analyzed by Chi-square test (α=0,05).

**Results:**

There was no difference in μTBS among the experimental groups. However, there was a statistically significant difference among 2.5 % nanofiber adhesive, 0.5 % nanofibers and control groups, (*p*=0,028) in relation to nanoleakage.

**Conclusions:**

TiO2 nanofibers in 2.5% of weight inserted in dental adhesive reduced the nanoleakage, but did not improve the μTBS to dentin.

** Key words:**Dentin-bonding agents, nanoleakage, tensile bond strength.

## Introduction

The advances in dental materials and adhesive technology have enabled the dentists to make esthetic anterior restorations in a simple and economical way (1). Nowadays, adhesive systems are widely used in direct procedures as restoration of anterior and posterior cavities, fissure sealing, reattachment of fractured fragments, corrections in tooth morphology and in indirect procedures involving cementation of root-canal posts and indirect ceramic and composite crowns (2).

Simplified etch-and-rinse adhesives have reduced clinical steps, but they have showed permeability of water from the oral environment and from the underlying bonded dentin (3-6), leading to incompatibility issues (7-9), faster degradation of resin-dentin bonds and may not be as durable as was previously assumed (10,11). The loss of bond strength and adhesive quality has mainly been attributed to degradation of the hybrid layer at the dentin-adhesive interface and deterioration of the dentin collagen fibrils. Numerous publications have demonstrated the lack of bond stability (12-15).

Different laboratorial approaches have been proposed to improve monomer infiltration, reduce the rate of water sorption, reduce collagen degradation and qualify the adhesion. Effects of primer/adhesive placement agitation and drying time for five seconds on dentin have showed improvement of the shear bond strength to dentin (16). Another method to improve gear to qualify the adhesion is the use of a warm air-dry stream after primer application, because this technique reduces the nanoleakage (17). The technique can also be used to improve the mechanical and biological properties of universal adhesive systems (18,19).

Thinking about biomaterials, pure TiO2 nanofiber is being used in tissue engineering applications as polymeric scaffolds, to drive cell differentiation and create an osteogenic environment without the use of exogenous factors (20). The nanofibers that are fabricated by an electrospinning method show excellent antimicrobial activity against gram-negative Escherichia coli and gram-positive *Staphylococcus aureus* (21). Its antibacterial potential makes titanium dioxide an interesting choice to be incorporated into adhesive systems, specially that in the total etch approach, because they remove the smear layer completely and expose the collagen fibers, but it is not known if the titanium dioxide incorporation would influence the adhesive’s properties.

TiO2 nanofibers structure are successfully prepared by electrospinning technique followed by calcination process and should act as cross-linking agents in adhesive systems, also, this nanoparticles are increasingly being used in pharmaceutical, medical and cosmetic products (22). The TEM and XRD analyses show that TiO2 has uniform diameter of around 200 nm, and their length to width aspect ratio ranged between 5 and 15 (23).

The aim of this study was to evaluate the incorporation of TiO2 nanofiber in an ethanol-based adhesive by means of microtensile bond strength (μTBS) and nanoleakage. The hypothesis is that the performance of a total etch adhesive system with and without titanium dioxide nanofibers will be similar.

## Material and Methods

-Experimental design

It was an *in vitro* study involving the incorporation of titanium dioxide nanofibers in an ethanol-based total etch adhesive systems (in four levels). Bond strength and nanoleakage were the main response variables for comparison purposes.

-Specimen preparation

Twenty extracted, caries-free human third molars were used in this study. The teeth were disinfected in 0.5% chloramine, stored in distilled water and used within 6 months after extraction. A ﬂat dentin surface was exposed after wet grinding the occlusal enamel on a # 180 grit SiC paper. The exposed dentin surfaces were further polished on wet #600-grit silicon-carbide paper for 60 seconds to standardize the smear layer.

One solvent-based, etch-and-rinse adhesive systems without inorganic particles were tested: Ambar ® (FGM, Joinvile, SC, Brazil). All the inorganic nanofibers were prepared by sol-gel processing and electrospinning technique using a viscous solution (chemistry department, Federal University of Rio Grande do Sul), and it has an antimicrobial function as well. Each nanofiber has about 200 to 300 nanometers of diameter. The adhesive received a nanofiber insert by weight, at 0.5, 1.5 and 2.5%, it is forming three groups respectively (G1, G2 and G3). The control group was the original bonding agent – G4.

After acid etching, the surfaces were rinsed with distilled water for 15 s and air-dried for 15 s. The surfaces were then rewetted with water (24). Two coats of adhesive were gently applied for 10 s. After each coat, the solvent was evaporated (distance: 20cm) to performed it function. The adhesive were light-cured for the respective recommended time using a LED 1200 mw/cm2 (Radii cal, SDI, Australia). Resin composite build-ups (Z350 XT, Shade A2 Body, 3M ESPE, St. Paul, MN, USA.) were constructed on the bonded surfaces in 3 increments of 1 mm each that were individually light-cured for 20 seconds with the same light intensity. All the bonding procedures were carried out by a single operator at a room temperature of 20°C and constant relative humidity. All the teeth were stored in distilled water at 37°C for 24 h.

-Bond strength test

The restored teeth were longitudinally sectioned perpendicularly to the bonded interfaces with a diamond saw (Isomet 1000, Buehler, Ilinois, USA) to obtain the sticks. Specimens areas were measured with a digital caliper and recorded (Absolute Digimatic, Mitutoyo, Tokyo, Japan). Each stick had a cross-sectional area of approximately 0.96 mm2.

Half of the specimens, from each tooth, were tested in microtensile testing, and they were randomly assigned. The other half was analyzed by SEM, in relation to nanoleakage. Each bonded stick was attached to a device for microtensile bond strength test (μTBS), with cyanoacrylate resin (Zapit, Dental Ventures of North America, Corona, CA, USA) and subjected to a tensile force in a Microtensile tester (Bisco, USA) at a crosshead speed of 0.5 mm/min (n=25).

The mean BS for every testing group was expressed as the average of the ﬁve teeth used per group. The micro tensile bond strength data was subjected to one-way analysis of variance, with a significant level set at 0.05.

-Nanoleakage evaluation

This study utilized ten sticks from each group for nanoleakage evaluation (n=40). None of the sticks evaluated by nanoleakage were tested by μTBS. The sticks were coated with two layers of fast-setting nail varnish applied up to within 1 mm of the bonded interfaces. The specimens were re-hydrated in distilled water for 10 min prior to immersion in the tracer solution. Conventional silver nitrate (Sigma Chemical Co. St. Louis. MO; USA) was prepared in a 50 wt% silver nitrate solution (pH=4.2) The sticks were placed in the conventional silver nitrate in darkness for 24 h, rinsed thoroughly in distilled water, and immersed in photo developing solution for 8 h under a ﬂuorescent light to reduce silver ions into metallic silver grains within voids along the bonded interface.

All these sticks were wet-polished with 600-grit SiC paper to remove the nail varnish. Then, the specimens were placed inside an acrylic ring, which was attached to a double-sided adhesive tape, and embedded in epoxy resin. After the epoxy resin set, the thickness of the embedded specimens was reduced to approximately half by grinding with silicon carbide papers under running water. Specimens were polished with a 600, 1000, 2000 and 2400 grit SiC paper and 6, 3, 1 and 0.25 mm diamond paste (Buehler Ltd., Lake Bluff, IL, USA) using a polish cloth. They were ultrasonically cleaned, silica dried, mounted on stubs, and coated with carbon-gold (MED 010, Balzers Union, Balzers, Liechtenstein). Resin–dentin interfaces were analyzed in a ﬁeld-emission scanning electron microscope operated in the backscattered electron mode (JSM 5800, JEOL, Tokyo, Japan). Presence or absence of nanoleakage was observed, and this data were analyzed by Chi-square test, with a significant level set at 0.05.

## Results

-Bond Strength Test (µTBS)

The range of cross-sectional area was 0.96mm2 for all sticks. The different values of original adhesive (G4) and modified adhesives (Titanium dioxide nanofiber insert by weight) G1, G2, G3 respectively are summarized in Table 1. One-way ANOVA revealed that there were no statistically significant differences among the groups (*p*=0,607).

**Table 1:**
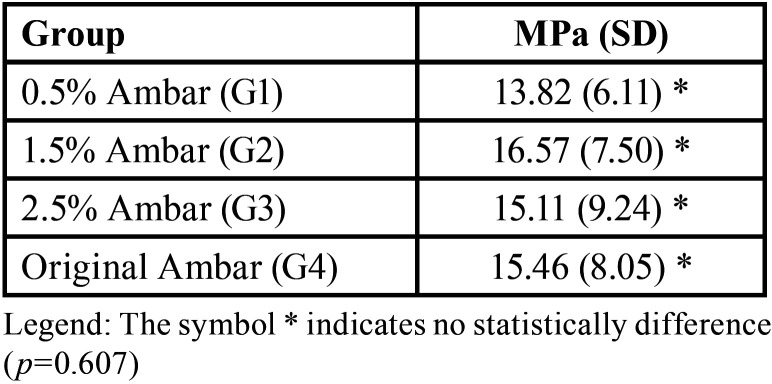
Bond strength, in MPa (standard deviations) of experimental groups.

-Nanoleakage evaluation

The presence or absence of nanoleakage was noted and it is shown in Table 2. Data were analyzed by Chi-square test, with significance level set at 5%. There was a statistically significant difference among group 3 (2,5 % titanium dioxide nanofibers) and groups 1 and 4 (0,5 % nanofibers and control, respectively) (*p*=0,028).

**Table 2:**
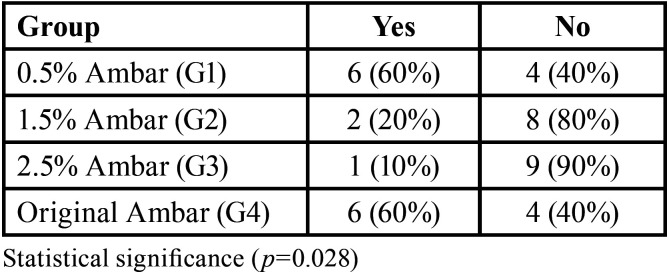
Number (percentages %) of samples with (YES) and without (NO) nanoleakage after Chi-square test.

**Figure 1:**
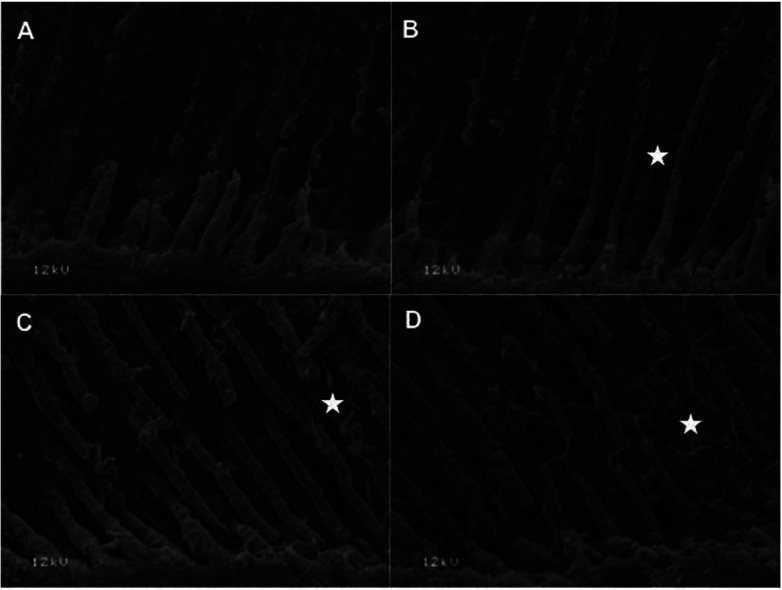
Representative backscattered SEM images of resin-tag morphology according to the amount of titanium dioxide nanofibers (A-0%; B-0.5%; C-1.5%; D-2.5%). The amount of crosslink nanofibers (white star) increases directly to the percentage of titanium dioxide incorporation.

**Figure 2:**
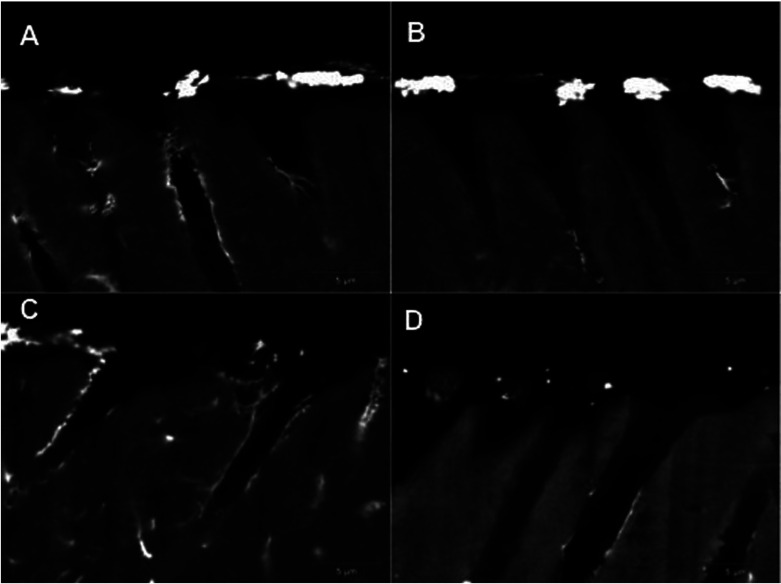
Representative backscattered SEM images of the resin-dentin interfaces of a total etching adhesive system with different percentages of titanium dioxide nanofibers (A-0%; B-0.5%; C-1.5%; D-2.5%). The silver nitrate deposites inside the adhesive layer (in white) decreases as the amount of titanium dioxide increases from A to D.

Representative SEM backscattered images of the resin tags (Fig. 1) and resin-dentin interfaces (Fig. 2) of all groups were obtained. In general, the amount of silver nitrate decreased with the increased incorporation of titanium dioxide nanofibers (Fig. 2, white areas). The higher silver nitrate depositon was observed at control and 0.5% nanofibers groups while less nanoleakage was observed at 1.5 and 2.5% of fibers.

Regarding the resin-tags, Figure 1 shows higher amount of crosslink of nanofiber with tags in dentin as the amount of titanium dioxide increased. As observed for nanoleakage, groups 1.5 and 2.5% of titanium dioxide showed higher cross-linking nanofibers than that of 0.5% group. The control group (without nanofiber incorporation) did not show nanofibers within the resin tags.

## Discussion

In the current study, the microtensile bond strength of adhesive systems was compared using different amount of nanofibers in adhesive without filled, manufactured by FGM (FGM, Joinvile, SC, Brazil). We believe that the microtensile testing and nanoleakage analysis are convenient methods for screening and testing the strength and quality of adhesive interfaces *in vitro* (2,9).

The higher performance of this kind of actual adhesives has been previously reported and they are used to make esthetic anterior restorations in all patients (1). The aim of this study was trying to improve the adhesion in dentin, based on quality in adhesion and if its possible, more strength to hybrid layer and more stability (4,5).

The adhesive used in the current study was intentionally chosen because it is not filled with inorganical particles. Although the incorporation of titanium dioxide nanofibers did not improve the bond strength of Ambar, it did not decrease its performance. The hypothesis must be accepted in terms of bond strength measurement.

However, the application of the nanofibers in adhesive system significantly affected the quality of hybrid layer in relation to nanoleakage. The incorporation of titanium dioxide nanofibers at 1.5 and 2.5% levels resulted in the lowest levels of nanoleakage of the adhesive system when compared to the original bonding agent without nanofibers. For this reason, the hypothesis must be accepted, since differences were observed among the groups. It was also noted the presence of nanofibers with crosslink with tags at the same groups, and the collagen probably. If this really occurs, it is important to analyses the durability of bonding in these adhesive compositions, because the crosslink is a form to stabilize the composite adhesive and the TiO2 nanofibers are insoluble in water, and this fact can also contribute to reduce the nanoleakage (9,10). The percentage of silver nitrate penetration in interfacial space was significantly higher in the specimens without nanofibers. It is possible to believe that the nanofibers were inserted in chemical crosslink polymer in an ideal amount, a reduction of the composite resin shrinkage would occur (25).

This current study verified that the resin-dentin bond strength values were similar for those different amounts of nanofibers. This finding could be attributed to the fact that the same material was applied to build the nanofiber. The high performance of the hybrid layer formed without nanoleakage in almost all specimens was probably because the nanofiber net reduced the polymerization stress, polymerization shrinkage and elastic modulus (26). According to Ozel and Soyman (25), composites that received fiber nets showed significantly lower microleakage and stress decrease, and this study showed this result using a qualitative analysis by nanoleakage.

The tags penetration was not influenced by nanofibers in every test groups, view by scanning electronic microscopy. Unfortunately, this issue has not yet been evaluated in other adhesives ethanol and alcohol based and should be further investigated. We believed that this paper is the first step to open the doors for titanium dioxide nanofibers in adhesive systems; however, clinical trials must be conducted to verify these results in a clinical situation.

## Conclusions

Within the limitation of this study, it was possible to conclude that titanium dioxide nanofibers in 2,5% of weight inserted in a dental bonding agent reduced the nanoleakage within the hybrid layer, and did not improve the μTBS of composite restorations.
